# Stent-Assisted Coiling for a Ruptured Wide-Neck Basilar Artery Trunk Aneurysm With Preservation of Brainstem Perforators: A Case Report

**DOI:** 10.7759/cureus.105889

**Published:** 2026-03-26

**Authors:** Yuya Ogura, Shin Nemoto, Ririko Takeda, Hajime Nishido, Makoto Nakane

**Affiliations:** 1 Department of Neurosurgery, Teikyo University Mizonokuchi Hospital, Kawasaki, JPN; 2 Department of Neurosurgery, Teikyo University Chiba Medical Center, Ichihara, JPN

**Keywords:** basilar artery trunk aneurysm, dual antiplatelet therapy, endovascular treatment, stent-assisted coiling, subarachnoid hemorrhage

## Abstract

Basilar artery trunk aneurysms are rare, accounting for 2.1% of all intracranial aneurysms. Among these, Mizutani type 4 (nonbranching true aneurysms) carries a particularly high risk of rupture and rebleeding. Although endovascular therapy is common, reports on the safety and efficacy of stent-assisted coiling (SAC) for ruptured wide-neck basilar artery trunk aneurysms in the acute phase remain limited. A 60-year-old man presented with subarachnoid hemorrhage (World Federation of Neurosurgical Societies grade I). Imaging revealed a wide-neck saccular aneurysm (4.8 mm dome diameter, 5.4 mm neck width) in the basilar artery trunk, consistent with a Mizutani type 4 aneurysm. On day 2, SAC was performed using a Neuroform Atlas stent. Because the patient required no additional invasive procedures like external ventricular drainage, dual antiplatelet therapy (DAPT) was initiated immediately before the procedure. Complete occlusion was achieved while maintaining the patency of the brainstem perforators. The patient was discharged with a modified Rankin Scale score of 0, and stable occlusion was maintained at the three-month follow-up. In conclusion, SAC using the Neuroform Atlas stent on DAPT is a viable treatment option for ruptured wide-neck basilar artery trunk aneurysms. This approach allows for secure aneurysm occlusion while minimizing the risk of ischemic complications in the perforator-rich basilar trunk.

## Introduction

Basilar artery trunk aneurysms are rare, accounting for 2.1% of all intracranial aneurysms [1]. Their pathology can be broadly divided into true aneurysms and arterial dissections. Among them, Mizutani type 4 (nonbranching true aneurysms) is associated with a high risk of rupture and rebleeding [2,3]. In addition, because this region lies deep within the cranial cavity and contains dense brainstem perforators, treatment planning requires careful decision-making [4]. The optimal management of basilar artery trunk aneurysms remains controversial because microsurgical clipping is technically demanding due to their deep location and the need to preserve perforators. At the same time, endovascular therapy (EVT) requires careful antiplatelet management and raises concerns about long-term durability.

Although EVT has become widely used, reports on the efficacy and safety of stent-assisted coiling (SAC) for ruptured wide-neck basilar artery trunk aneurysms remain limited [5,6]. In recent years, novel endovascular devices such as flow diverters (FDs), intrasaccular flow disruptors, and overlapping stents have emerged as promising treatment options [7-9], particularly for unruptured complex aneurysms. However, their use in the acute phase of rupture remains limited due to concerns regarding antiplatelet therapy and hemorrhagic complications. Here, we report a case of a ruptured wide-neck basilar artery trunk aneurysm treated with SAC with a favorable clinical course, along with a review of the relevant literature.

## Case presentation

The patient was a 60-year-old Japanese man with a history of well-controlled type 2 diabetes mellitus. He had no notable family history. On the day of symptom onset, he developed dizziness and vomited three times, and he walked to a local clinic for evaluation. A non-contrast head CT at the clinic demonstrated subarachnoid hemorrhage (SAH), and he was urgently transferred to our hospital the same day.

On arrival, his level of consciousness was Glasgow Coma Scale (GCS) score E4V5M6, and he had no focal neurological deficits such as motor weakness or aphasia. He was classified as World Federation of Neurosurgical Societies (WFNS) grade I. 

**Figure 1:**
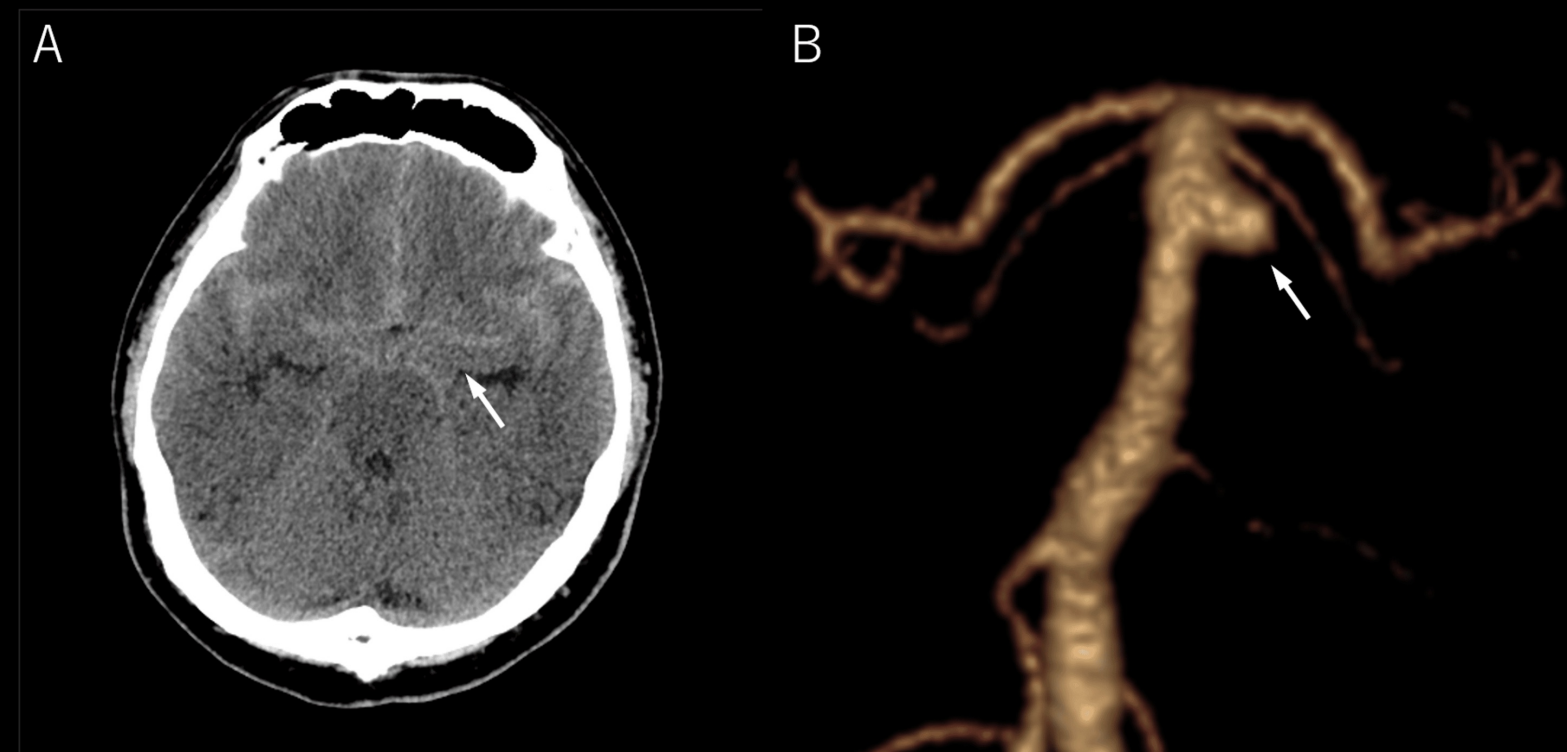
Head CT and three-dimensional CTA (on admission) Head CT revealed diffuse SAH (Fisher Group III) (arrow) (A). Three-dimensional CTA with VR CTA revealed that the aneurysm is located at the basilar artery trunk (arrow). The basilar artery trunk aneurysm was measured 4.8 × 4.6 mm in maximum diameter and the neck width was 5.4 mm (B). CTA, computed tomography angiography; SAH, subarachnoid hemorrhage; VR, volume rendering.

On admission, non-contrast head CT showed diffuse SAH, corresponding to Fisher group 3 (Figure 1A). Subsequent CT angiography (CTA) demonstrated an aneurysm arising from the basilar artery trunk, measuring 4.8 × 4.6 mm in maximum diameter (Figure 1B). The neck width was 5.4 mm, with a dome-to-neck (D/N) ratio of 0.88.

**Figure 2:**
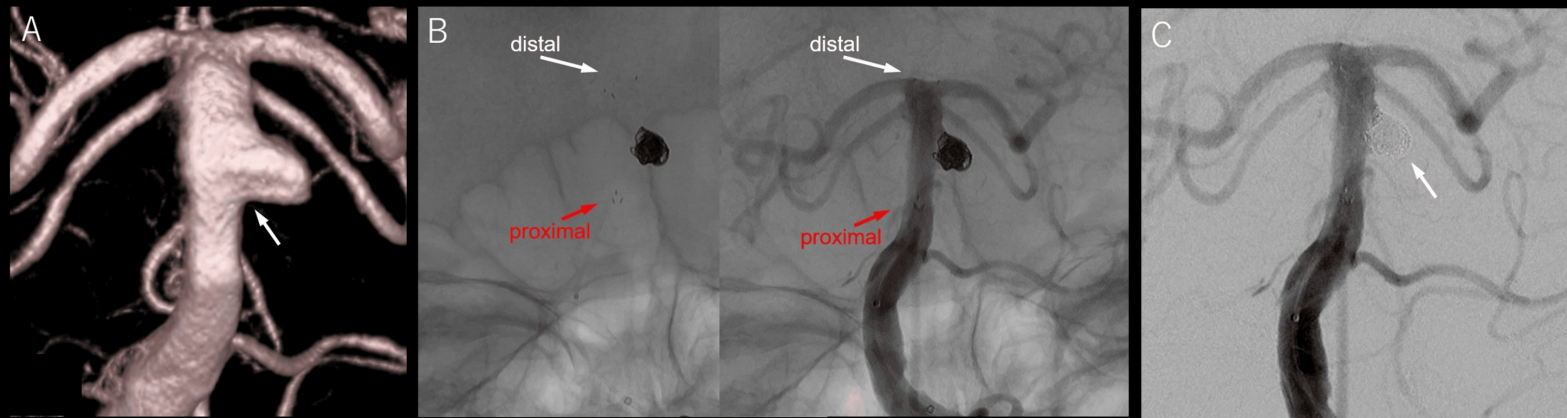
Intraoperative DSA Preprocedural 3D angiographic reconstruction revealed an aneurysm in the basilar trunk artery. No perforating branches were identified arising near the aneurysmal neck (arrow) (A). A Neuroform Atlas stent was deployed from the basilar artery tip (white arrow) to the basilar artery trunk to cover the neck (red arrow) (B). Postprocedural DSA showed patency of the parent artery and complete occlusion of the aneurysm (arrow) (C). DSA, digital subtraction angiography.

CTA showed no definite findings strongly suggestive of dissection, such as marked irregular stenosis of the parent vessel, and short-term follow-up imaging revealed no change in aneurysm morphology. In addition to its morphological characteristics as a wide-neck saccular aneurysm arising from a non-branching segment, the intraoperative DSA findings also suggested that the lesion was more consistent with a true aneurysm (Mizutani Type 4) rather than a dissecting aneurysm (Figure 2A).

Endovascular treatment was selected due to the deep location of the aneurysm and the presence of critical perforators, which made microsurgical clipping technically challenging. We considered that stable framing would be difficult with simple coiling or balloon assistance alone because this was a wide-neck aneurysm. Therefore, we selected SAC. As perioperative antiplatelet therapy, aspirin (200 mg) and prasugrel (20 mg) were administered as a loading dose immediately before the procedure.

Under general anesthesia, an 8-Fr FUBUKI guiding catheter (Asahi Intecc, Aichi, Japan) was inserted through the right common femoral artery into the left vertebral artery, and a 6-Fr FUBUKI guiding catheter (Asahi Intecc) was inserted through the left common femoral artery into the right vertebral artery. A Scepter XC balloon catheter 4 × 11 mm (Terumo, Tokyo, Japan) was prepared for deployment in case of intraoperative rupture from a 6-Fr FUBUKI guiding catheter (Asahi Intecc) through the right vertebral artery. Initially, for stenting, an SL-10 microcatheter (Stryker Neurovascular, Fremont, CA, USA) was inserted into the left posterior cerebral artery (PCA). Subsequently, for the intra-aneurysmal approach, a Greach 90-5 microcatheter (Tokai Medical Products, Aichi, Japan) was navigated into the aneurysm coaxially with a Guidepost (Tokai Medical Products). A Neuroform Atlas stent (4.5 × 21 mm) was deployed from the basilar artery tip to cover the neck.

As the first coil, a Target 360 ultrasoft 4.5 mm/10 cm (Stryker Neurovascular) was inserted using the jail technique. Subsequently, two OPTIMA complex SS 4 mm/8 cm (Balt Extrusion, Montmorency, France) and two i-EDCOIL complex SS 2 mm/4 cm (Kaneka Medix Corporation, Osaka, Japan) were inserted. For finishing, TARGET tetra 2 mm/2.5 cm (Stryker Neurovascular) was inserted, and digital subtraction angiography (DSA) was performed to ensure that no blood flow was observed in the aneurysm and there was no protrusion into the stent (Figure 2B, 2C). The procedure was successful and did not cause any complications.

Because the patient had no acute hydrocephalus on admission and there were no findings requiring external ventricular drainage, no additional invasive procedures were performed, and dual antiplatelet therapy (DAPT; aspirin 200 mg and prasugrel 3.75 mg) was continued throughout the perioperative period. The postoperative course was uneventful. MRI obtained the next day showed no new ischemic lesions, and no focal neurological deficits were observed. The patient was discharged home on postoperative day 25 with a modified Rankin Scale (mRS) score of 0. Three-month follow-up MRA showed no evidence of recurrence or in-stent restenosis.

## Discussion

Basilar artery trunk aneurysms are rare vascular lesions, accounting for only 2.1% of all intracranial aneurysms. However, ruptured cases have been reported to be associated with substantial morbidity and mortality [10,11]. According to the classification proposed by Mizutani and colleagues, this region includes both dissecting aneurysms (types 1-3) and true aneurysms (type 4) [2].

In the present case, intraoperative DSA showed no morphological changes or irregular stenosis suggestive of dissection, and short-interval follow-up imaging demonstrated stable aneurysm morphology. These findings were considered consistent with a type 4 saccular aneurysm [2]. Given the risk of early rebleeding related to this intrinsic wall fragility [3], achieving secure aneurysm occlusion in the acute phase is critically important.

Surgical management of basilar artery trunk aneurysms is technically challenging because of the deep location and limited surgical access [3]. Adequate exposure often requires extensive skull base approaches, which carry risks of cranial nerve injury and complications related to brainstem manipulation. In addition, in wide-neck lesions such as the present case, microsurgical clipping may increase the risk of perforator injury and parent artery stenosis. For these reasons, we selected an endovascular approach. Furthermore, the aneurysm had an extremely wide neck (D/N ratio, 0.88), and simple coiling was considered to carry a high risk of coil protrusion into the parent vessel [12]. Therefore, we chose a reconstructive strategy using stent assistance.

The basilar artery trunk has a dense network of pontine perforators [13]. Recent advances in endovascular treatment have expanded treatment options for complex aneurysms, including FD and intrasaccular flow disruptors. When we select a device, FD provides strong flow-modifying effects. However, there is concern for ischemic complications when important perforators are covered [7,14].

In contrast, the Neuroform Atlas stent used in the present case has an open-cell design with a relatively low metal coverage (approximately 6%-12%) [15]. This feature may help minimize compromise of perforator flow while providing a scaffold that facilitates dense coil packing within the aneurysm [15,16]. No new ischemic lesions were observed on postoperative MRI.

Stent placement in the acute phase after aneurysm rupture requires initiation of DAPT to prevent thromboembolic events. In the present case, the absence of acute hydrocephalus on admission and the lack of need for additional invasive procedures such as external ventricular drainage allowed early initiation of DAPT [17-19]. A loading dose of aspirin (200 mg) and prasugrel (20 mg) was administered, and the patient was managed with close perioperative monitoring without major complications. At three months, the patient maintained a favorable neurological outcome (mRS 0), with persistent occlusion and stent patency.

Wang et al. reported a high treatment-related risk for EVT of basilar artery trunk aneurysms, with a complication rate of 35.7% and a mortality rate of 17.9% [6]. However, details of treatment protocols, including stent type and DAPT timing and duration, were not sufficiently described. Accumulating such protocol-level reports is therefore important. Our case provides one such example, achieving an mRS 0 after SAC with a Neuroform Atlas stent and initiation of DAPT immediately before the procedure.

As limitations of this report, it is based on a single case, and follow-up was limited to three months. Longer-term observation is required to assess long-term stent patency and the risk of recurrence after stent placement in this location.

## Conclusions

SAC using the Neuroform Atlas stent on DAPT may be a useful treatment option for ruptured wide-neck basilar artery trunk aneurysms, allowing prevention of rebleeding and aneurysm occlusion while minimizing compromise of brainstem perforator flow. Although there have been very few reports of SAC for these aneurysms in the literature, the accumulation of more cases such as the present one may gradually prove its effectiveness for this disease.
